# Supplementary material for: Years of Potential Life Lost on Renal Replacement Therapy: Retrospective Study Cohort
Source: J Clin Med. 2022 Dec 21;12(1):51. doi: 10.3390/jcm12010051 (PMC9821093; doi:10.3390/jcm12010051)
Supplement: Supplementary file 1 [file jcm-12-00051-s001.zip › jcm-1990739-supplementary.pdf]

Suplemental Materials

Table S1. Renal disease that motivated renal replacement therapy.

	N (%)	CI 95%
**Diabetic Nephropathies**	2832 (24,5)	(23,7; 25,3)
**Glomerular disease**	1747 (15,1)	(14,5; 15,8)
**Hypertension and renovascular disease**	1684 (14,6)	(13,9; 15,2)
**Renal tubulo-interstitial nephropathy**	1222 (10,6)	(10,0; 11,1)
**Familial / hereditary nephropathies**	971 (8,4)	(7,9; 8,9)
**Systemic diseases affecting the kidney**	389 (3,4)	(3,1; 3,7)
**Miscellaneous renal disorders**	305 (2,6)	(2,4; 2,9)
**Chronic renal failure; aetiology uncertain**	2401 (20,8)	(20,1; 21,5)

Table S2. Comorbidities of patient included in the cohort of renal replacement therapy.

Comorbidities	N (%)	IC 95%
Diabetes mellitus	4443 (38.5)	(37.6; 39.4)
Congestive heart failure	2184 (18.9)	(18.2; 19.6)
Myocardial infarction	2066 (17.9)	(17.2; 18.6)
Peripheral vascular disease	1736 (15.0)	(14.4; 15.7)
COPD	1406 (12.2)	(11.6; 12.8)
Solid tumor localized	1031 (8.9)	(8.4; 9.5)
Cerebrovascular accident	983 (8.5)	(8.0; 9.0)
Connective tissue disease	476 (4.1)	(3.8; 4.5)
Peptic ulcer disease	372 (3.2)	(2.9; 3.6)
Mild Liver disease	388 (3.4)	(3.0; 3.7)
Moderate to severe liver disease	195 (1.7)	(1.5; 1.9)
Leukemia/Lymphoma	175 (1.5)	(1.3; 1.8)
Dementia	148 (1.3)	(1.1; 1.5)
Solid tumor metastatic	117 (1.0)	(0.8; 1.2)
VIH	96 (0.8)	(0.7; 1.0)
AIDS	23 (0.2)	(0.1; 0.3)

Table S3a. Potential years of life lost in men in RRT by age group

	2008	2009	2010	2011	2012	2013	2014	2015	2016	2017	2018	TOTAL
15-29 years	0,0	49,2	55,4	57,6	113,2	50,4	0,0	0,0	0,0	0,0	64,5	390,3
30-34 years	44,0	46,3	233,1	94,8	136,6	49,4	46,7	46,4	0,0	0,0	45,9	743,2
35-39 years	120,5	40,5	246,0	83,2	121,2	40,7	126,7	41,5	42,0	42,1	0,0	904,3
40-44 years	356,0	326,6	74,1	149,2	223,3	186,2	189,3	110,2	37,2	37,3	0,0	1689,5
45-49 years	600,5	294,4	319,8	351,2	355,7	463,9	202,0	291,1	101,3	192,0	102,2	3274,2
50-54 years	612,0	500,6	653,7	285,7	700,2	453,1	314,1	455,2	256,4	112,6	174,4	4517,8
55-59 years	842,9	629,6	711,4	408,7	511,7	433,7	587,8	461,6	247,7	495,1	120,4	5450,6
60-64 years	929,6	852,6	776,7	904,5	661,0	401,2	684,0	651,4	245,3	449,6	120,0	6675,9
65-69 years	747,2	1062,2	871,6	951,3	912,1	804,0	866,1	430,6	484,7	316,4	316,4	7762,8
70-74 years	856,2	919,1	803,0	863,8	636,1	712,4	511,8	716,3	509,5	459,9	193,7	7181,8
75-79 years	696,1	693,0	715,5	681,0	590,9	536,1	590,5	411,6	388,9	292,4	179,3	5775,4
80-84 years	247,3	300,0	295,8	237,5	337,2	342,3	355,6	329,5	297,3	181,5	101,3	3025,3
85-89 years	47,1	38,5	45,9	57,0	79,6	79,4	70,3	26,7	53,6	63,6	5,4	567,1
≥ 90 years	3,8	3,9	0,0	16,3	15,6	0,0	4,2	0,0	4,1	4,1	0,0	51,9

Table S3b. Potential years of life lost in women in RRT by age group

	2008	2009	2010	2011	2012	2013	2014	2015	2016	2017	2018	TOTAL
15-29 years	0,0	0,0	56,2	124,4	65,2	55,7	0,0	56,5	0,0	65,8	62,8	486,6
30-34 years	103,1	157,8	0,0	0,0	53,3	53,7	105,8	0,0	102,0	0,0	0,0	575,7
35-39 years	140,9	189,1	45,4	49,4	45,5	47,8	0,0	0,0	0,0	49,0	0,0	567,0
40-44 years	162,4	81,5	168,2	83,2	130,5	84,9	85,2	170,9	0,0	87,2	0,0	1053,9
45-49 years	250,6	39,3	110,4	111,4	113,2	185,8	40,2	116,6	111,2	0,0	38,3	1117,1
50-54 years	255,8	258,9	328,4	230,5	196,3	269,2	165,9	166,9	136,3	0,0	31,7	2039,9
55-59 years	501,4	469,0	361,7	275,2	310,4	321,3	200,7	259,7	257,2	85,8	54,2	3096,9
60-64 years	525,1	745,4	412,5	415,5	398,9	459,5	224,0	241,2	276,8	165,3	117,6	3981,9
65-69 years	641,0	547,6	531,6	655,6	380,0	418,5	474,6	212,7	259,2	185,9	164,4	4471,0
70-74 years	765,2	492,1	592,7	411,9	537,3	486,3	371,0	343,4	520,3	215,9	183,0	4919,0
75-79 years	537,5	581,3	742,6	603,9	466,5	379,3	428,3	338,7	338,6	187,5	90,3	4694,5
80-84 years	113,9	45,3	46,5	46,5	110,3	47,7	28,9	46,2	57,8	38,3	19,2	2296,9
85-89 years	97,7	67,6	34,6	69,3	68,5	62,3	44,9	51,5	62,8	44,6	8,9	495,2
≥ 90 years	37,8	39,4	64,4	32,3	31,9	57,9	50,2	55,7	16,7	33,1	8,3	25,7
